# Composition of Human-Associated Gut Microbiota Determines 3-DF and 3-HF Anti-Colitic Activity in IL-10 -/- Mice

**DOI:** 10.3390/nu16234232

**Published:** 2024-12-07

**Authors:** Jose Haro-Reyes, Jayaprakash Kanijam Raghupathi, Lavanya Reddivari

**Affiliations:** 1 Department of Food Science, Purdue University, West Lafayette, IN 47907, USA; jharorey@purdue.edu (J.H.-R.); jkanijam@purdue.edu (J.K.R.); 2 Department of Chemistry, Acharya Nagarjuna University, Guntur 522510, Andhra Pradesh, India

**Keywords:** flavonoids, anthocyanins, phlobaphenes, humanized mice, dysbiosis, gut barrier dysfunction, colitis

## Abstract

Background: Gut bacterial dysbiosis along with intestinal mucosal disruption plays a critical role in inflammatory disorders like ulcerative colitis. Flavonoids and other food bioactives have been studied in mice models as alternative treatments with minimal side effects. However, most of the research has been carried out with mice-native microbiota, which limits the comprehension of the interaction between flavonoids and human-associated bacteria. Hence, the objective of our study was to determine the effect of healthy human-associated microbiota on the anti-colitic activity of diets rich in anthocyanins (3-HF) and phlobaphenes (3-DF). Methods: In this regard, the interleukin (IL)-10 -/- mice model was utilized. Mice were divided into three groups for inoculation with human gut bacteria from three different healthy donors and assigned to four diets. A purified diet (Diet P) and three diets containing 25% near-isogenic lines (NILs) of corn were evaluated. Diets were substituted with NILs expressing only 3-DFs (diet B), only 3-HFs (diet C), and both 3-DF and 3-HF (diet D). Results: In an overall analysis, flavonoid-rich diets did not affect inflammatory markers, microbiota diversity, or gut metabolites, but diets containing anthocyanins improved barrier function parameters. However, when data was segmented by the recipient’s microbiota from different human donors, the diet effects became significant. Furthermore, 3-HFs showed more beneficial effects than 3-DFs across the recipient’s microbiota. Conclusions: Our study suggests that the anti-colitic activity of 3-DF and 3-HF and their gut metabolites depends on the donor’s microbial composition.

## 1. Introduction

Gut bacterial dysbiosis and an exacerbated immune response play a key role in the development of chronic inflammatory diseases such as ulcerative colitis (UC), a condition of inflammatory bowel disease (IBD) [1]. Gut bacterial dysbiosis is an imbalance in the microbiota composition, resulting in the prevalence of bacteria associated with disease status. These compositional changes induce or maintain existing inflammation and barrier dysfunction [2]. Furthermore, barrier dysfunction induced by congenic or environmental factors can potentially cause IBD [3]. The disease has become a public health problem in the US, where it affects more than 1% of the adult population [4]. Research has focused on multiple approaches to reduce gut inflammation and alleviate the disease with minimal side effects, like foods rich in bioactive compounds [5]. Dietary flavonoids have demonstrated effectiveness in ameliorating UC symptoms both in vitro and in vivo using human and animal models [6]. Different flavonoid-rich extracts or isolated compounds exhibited anti-inflammatory properties and resulted in higher levels of gut barrier structural proteins, short-chain fatty acids, and mucus thickness [6,7,8]. However, most of these studies utilized bioactives as plant extracts or isolated compounds, disregarding the natural interactions within their food matrix. Alternatively, the use of near-isogenic lines (NILs) allowed the assessment of specific flavonoid activity while maintaining their interactions with the food matrix [9]. Among flavonoids, 3-hydroxyflavonoids (3-HF) or anthocyanins and 3-deoxyflavonoids (3-DF) or phlobaphenes have demonstrated anti-inflammatory properties as evidenced by the reduction in IL-1β and IL-6, reduced gut barrier permeability, restored gut barrier integrity, and enhanced mucus thickness in different mice models [10,11]. Nevertheless, these studies involved UC mice models where dysbiosis was induced in the native microbiota of the mice. The potential effects of human-associated microbiota in the 3-DF and 3-HF activity remain unknown.

Colonization of the mice gut with human-associated microbiota involves multiple challenges. The efficiency of colonization is between 44 and 70%, which results in a different community from the human donor [12]. Moreover, humanized mice present an underdeveloped immune system [13] and a distinct metabolome [14], which can interfere with the anti-inflammatory response. Despite the differences and limitations, humanized mice models remain useful tools for observing ecological and functional responses to dietary treatments [12]. These models have shown that some associations found among bacteria and physiological responses can be translated to humans [15]. Germ-free IL-10 knockout mice transplanted with gut microbiota from pediatric UC patient fecal samples, which are prone to spontaneous colitis [16], were used in this study. These mice resemble IL-10 monogenetic disorders found in some human pediatric patients [17]. In addition, it is important to consider that inbred mice with the same genetic background express distinct colitis susceptibility with different microbiota profiles [18]. In consequence, it is reasonable to expect that human-associated microbiota can offer alternative results compared to previous studies with similar mice models.

Consequently, we hypothesize that the colonic anti-inflammatory activity of 3-DF and 3-HF, in the presence of human-associated microbiota in an IL-10 -/- mice model, will reveal distinctive microbiota compositional and functional dynamics. Understanding the relationships between bioactive compounds and specific microbes can contribute to the development of more effective dietary strategies for alleviating UC.

## 2. Materials and Methods

### 2.1. Plant Material and Diets

Three maize NILs (Penn State Russel Larson Agronomy Research farm, Rocsplings, PA, USA), with the expression of phlobaphenes and/or anthocyanins, were selected for the dietary treatments. Maize Line B expressed only phlobaphenes, Line C expressed only anthocyanins, and Line D contained both. Diets were based on AIN-93G diet composition with an inclusion of 25% DM from one of the maize lines, as shown in Table 1. The diets were balanced for energy, fat, and protein content. A purified diet (P diet) served as a control with no NILs. All the diets were manufactured by Envigo Teklad (Madison, WI, USA). Details on the genetic background, metabolite content, and development of the NILs were presented in our previously published article [9].

### 2.2. Experimental Design

**Figure 1:**
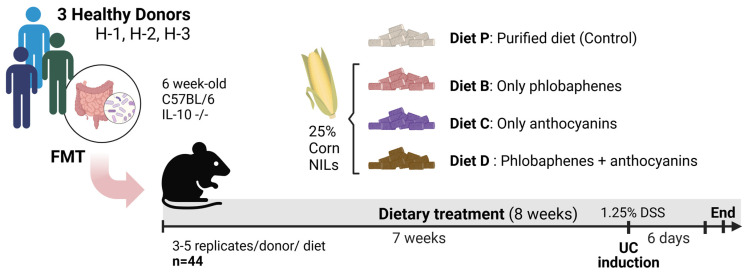
Experimental design. Fecal microbial transplantation (FMT), near-isogenic lines (NILs), ulcerative colitis (UC). Human fecal donors: H-1, H-2, and H-3.

All animal procedures were performed in accordance with Purdue University guidelines for animal care and use and experiments were approved by the Purdue Institutional Animal Care and Use Committee (protocol no. 1810001817). A staggered experiment was performed using 44 germ-free IL-10 -/- mice [C57BL/6] from Purdue University Animal Facility (West Lafayette, IN, USA). Mice underwent a one-week acclimation period prior to the start of the experiment, at which point they were six weeks old. They were then colonized via oral gavage with human fecal microbiota from healthy donors, all of whom had no history of chronic gastric diseases or active illness at the time of donation. Three to five mice per donor were randomly assigned to one of the experimental diets (P, B, C, and D), with nine to twelve mice per diet, for a total duration of eight weeks (Figure 1). In the final week, mice were administered 1.25% dextran sodium sulfate (DSS) in drinking water for six days to induce colonic inflammation. On the seventh day, mice were euthanized by CO_2_ asphyxiation. Blood samples were collected by cardiac puncture. Organs including the colon, cecum, liver, kidneys, and spleen were harvested and weighed, then processed or stored immediately for later use. Fecal content from the colon and cecum was collected and stored at −80 °C.

### 2.3. mRNA Expression of Colonic Biomarkers

The mRNA expression of pattern recognition receptors (PRRs), inflammatory cytokines, nuclear transcription factors, tight junction proteins, and mucin 2 were analyzed. Total RNA from frozen distal colon tissues was extracted using PureLink Mini Kit (Invitrogen, Carlsbad, CA, USA). RNA sample quantification was performed in a Take-3 plate with a BioTek™ Cytation™1 Cell Imaging Reader (Thermo Fisher Scientific Inc., Winooski, VT, USA). A concentration of 100 ng RNA/μL was employed for cDNA reverse transcription with the High-Capacity cDNA Reverse Transcription Kit (Applied Biosystems, Waltham, MA, USA), according to the manufacturer’s instructions. The Real-time PCR amplification and detection were performed on the QuantStudio 3 PCR instrument with PowerUp^TM^ SYBR^TM^ Green Master Mix (Applied Biosystems, Waltham, MA, USA) using respective primers (Table 2). The reaction consisted of an initial denaturation step at 95 °C for 10 min, followed by 45 cycles of 95 °C for 10 s annealing temperature with an extension step for 60 s at 55 °C. The mRNA expression was normalized using β-actin.

### 2.4. Disease Activity Index (DAI)

DAI was calculated following a previously published method [19] with modifications. In brief, after the DSS treatment, four parameters were evaluated. DAI was obtained by adding the individual scores of weight loss (0–3, 0 to >20% loss), stool consistency (0–3, normal to watery diarrhea), rectal bleeding/blood in the stool (0–3, no to severe bleeding), and mice condition (0–1, normal to poor condition).

### 2.5. Intestinal Permeability

The intestinal permeability was assessed with Fluorescein Isothiocyanate-Dextran (FITC-dextran) (Sigma-Aldrich, St. Louis, MO, USA). Briefly, mice were fasted for 4 h before euthanasia. After two hours of fasting, they were gavaged with 0.6% body weight (*w*/*v*) of 100 mg/mL FITC-dextran solution. Blood was collected by heart puncture into BD Microtainer^®^ tubes (BD, Franklin Lakes, NJ, USA). The fluorescence intensity was quantified [λex = 490 nm; λem = 520 nm] in a BioTek™ Cytation™1 Cell Imaging Reader (Thermo Fisher Scientific Inc., Winooski, VT, USA), and the intestinal permeability was calculated by standard curve.

### 2.6. Gut Microbiota 16S rRNA Gene Sequencing, Diversity and Composition

DNA was extracted from mice cecal contents using a QIAmp PowerFecal Pro DNA Kit (QIAgen, Germantown, MD, USA) following the manufacturer’s instructions. DNA concentration was quantified using a Take3 microvolume plate (BioTek, Winooski, VT, USA) in a BioTek™ Cytation™1 microplate reader (Winooski, VT, USA). DNA extracts were processed in the RUSH Genomics and Microbiome Core Facility (RUSH University, Chicago, IL, USA). The hypervariable V4 region was amplified (515F-806R) and sequenced on the Illumina MiniSeq Platform (Illumina, Inc., San Diego, CA, USA). Demultiplexed sequences were merged using the pair-end read merger (PEAR) version 0.9.11 and analyzed in the Quantitative Insights into Microbial Ecology2 (Qiime2) Platform version 2022.18. DADA2 pipeline was harnessed to filter and denoise single-end sequences. Trimming and truncating were set at 19 and 273 base pairs, respectively. Given the DADA2 and alpha-rarefaction outputs, a sampling depth of 15,288 was defined before calculating the diversity metrics. Taxonomy assignment was performed at the family level using SILVA 138 SSU 99% identity 16S 515 F/806 R database as a reference. Alpha-diversity was estimated in QIIME2 through four indexes: Shannon, Pielou, Faith_PD, and observed features. Beta-diversity was measured by Jaccard, Bray-Curtis, and unweighted and weighted UniFrac indexes. Only bacteria with a relative abundance higher than 1% were presented in the taxa-bar plots.

### 2.7. Short-Chain Fatty Acid Extraction and Quantification

Cecal content from each mouse was used for short-chain fatty acid (SCFA) extraction. Briefly, about 50 mg of cecal content was transferred to reinforced tubes with silica beads (Fisher Scientific, Hampton, NH, USA). A solution of 0.5% phosphoric acid was added to a ratio of 1:12 (*v*/*w*) of cecal content. Samples were homogenized using Bullet Blender Gold (Next Advance, Troy, NY, USA) set at power eight for a two-minute cycle, followed by a one-minute vortex. Homogenates were centrifuged at 17,000× *g* for 10 min at 4 °C. The supernatants were removed and mixed in a 1:1 (*v*/*v*) ratio with ethyl acetate solution containing heptanoic acid as an internal standard. The mixture was vortexed for two minutes, centrifuged and the supernatant (ethyl acetate phase) was used for SCFA quantification.

Acetate, propionate, and butyrate standards were purchased from Sigma-Aldrich (St. Louis, MO, USA). The standard curve was built for a mixture of the three SCFAs and IS at the following concentrations: 3, 1.5, 0.75, 0.375, and 0.1875 mM. Quantification was performed with a silica capillary column (Nukon supelco No. 40369-03A, Bellefonte, PA, USA) in an Agilent 7890A gas chromatogram system (GC-FID 7890A, Santa Clara, CA, USA). Chromatographic analysis conditions are explained in Supplementary Method S1.

### 2.8. Bile Acids Extraction and Quantification

Bile Acids (BAs) were extracted from each mouse’s blood serum as previously described [20] with modifications. Briefly, 50 µL of serum was mixed with 150 µL of cold methanol, containing 0.4 µg/mL of glycocholic acid as an internal standard (GCA-IS). The mixture was maintained at −20 °C for 20 min and centrifuged at 14,000× *g* for 20 min at 4 °C. The supernatant was used for analysis.

Targeted primary conjugated bile acids (glycocholic acid, GCA, taurochenodeoxycholic TCDCA, and tauroursodeoxycholic acid, TUDCA,) and unconjugated bile acids (Cholic acid, CA, Chenodeoxycholic acid, CDCA, α-murocholic acid, αMCA, and β-murocholic acid, β-MCA) were quantified using an Agilent 1260 Infinity II HPLC system coupled with an Ultivo triple quadrupole mass spectrometer. The analytical method was conducted in negative MRM mode to enhance sensitivity for targeted bile acid quantification. The HPLC gradient was adapted from existing methods with specific modifications [21]. The mobile phase comprised solvent A (20 mM ammonium acetate with 0.1% (*v*/*v*) formic acid) and solvent B (methanol). The analytes were analyzed using a Supelco Ascentis Express C18 column (150 × 4.6 mm, 2.7 μm) maintained at 40 °C. Mass spectrometer settings were optimized as follows: gas temperature at 200 °C, gas flow at 12 L/min, nebulizer pressure at 40 psi, sheath gas temperature at 200 °C, sheath gas flow at 10 L/min, and capillary voltage set to 3000 V. Data acquisition was performed in customized negative mode, and targeted MRM transitions were utilized for precise compound identification.

### 2.9. Statistical Analysis

Data were analyzed in two different ways for treatment comparison, within each donor or pooling all the donors together per treatment. All the data except the bacterial sequencing was analyzed with IBM SPSS Statistics 29.0 and graphed with GraphPad Prism 10. The normality assumption was tested using the Shapiro–Walk test. If the assumption was accepted, one-way ANOVA was applied with a DCA model, where the diets were assigned as treatments. Treatment means were compared with Tukey’s test, or the Games–Howell test when homogeneity of variances was refused by Levene heterogeneity test. When outliers were identified, they were removed, but if non-normality persisted, non-parametric Kruskal–Wallis or Mann–Whitney were applied. Microbial sequencing data was analyzed with R Studio version 2024.04.0 or Qiime2 and graphed with GraphPad Prism 10. Alpha-diversity data was tested with Kruskal–Wallis pairwise analysis while β-diversity was tested with pairwise PERMANOVA. Compositional differences were tested with ANCOM-BC. Spearman correlation was applied to analyze associations between bacteria taxa and other biomarkers evaluated. Significant correlations with r < 0.4 were described as mild, 0.4 < r < 0.6 as moderate, and r > 0.6 as high. Overall, statistical significance was indicated by: # = *p* < 0.10, * = *p* < 0.05.

## 3. Results

Our analysis, pooling the data from all donor recipients, revealed that the flavonoid-rich diets significantly changed a small portion of the parameters evaluated in this study. Interestingly, the limited response to the flavonoid-rich diets was primarily attributed to the microbiota of one or two human donor recipients’ groups and is not a pattern found in the whole set of donors. However, the effectiveness of diets and flavonoids differed, being effective, ineffective, or non-significant, depending on the donor’s microbiota. The results are explained through both approaches.

### 3.1. Anatomical and Symptomatic Changes After Colitis Induction

**Figure 2:**
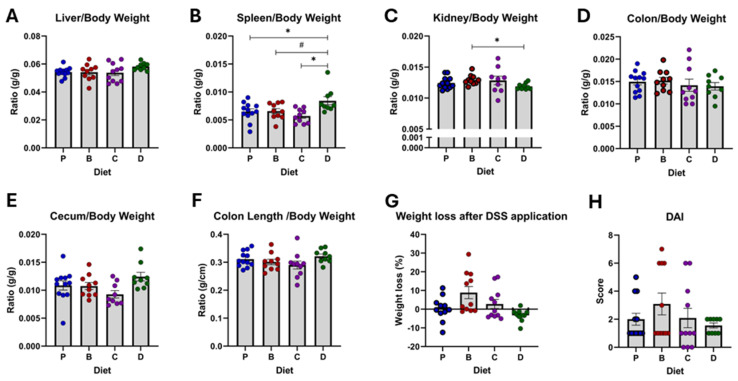
Overall diet effects on anatomic and symptomatic parameters. Liver (**A**), spleen (**B**), kidneys (**C**), colon (**D**), cecum (**E**), and colon length (**F**) are expressed as ratios of body weight, together with the percentage of weight loss (**G**) and disease activity index (**H**). Diets: purified (P), and 25% inclusion of maize NIL with 3-DFs (B), 3-HFs (C), or both 3-DF + 3HF (D). Replicates (*n* = 9–12) are shown for every diet. Statistical differences are represented by (*) *p* ≤ 0.05, and (#) *p* ≤ 0.10.

Flavonoid-rich diets did not affect food intake, organ size, weight loss, or DAI compared to the purified diet (P) (Figure 2A–H; Supplementary Figures S4 and S5). However, mice on diet D, which contained both 3-DF and 3-HF, had increased spleen weight (Figure 2B) compared to other diets and reduced kidney size compared to the 3-DF group (Figure 2C).

**Figure 3:**
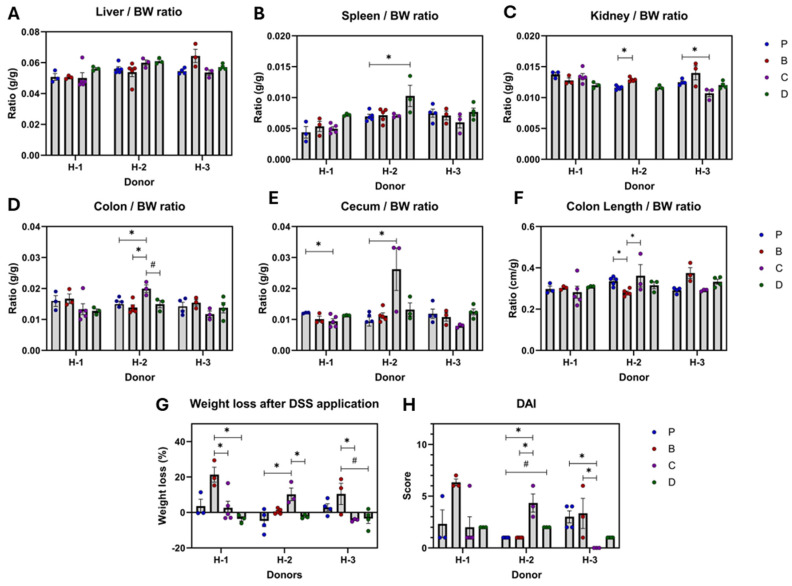
Diet effects on anatomic and symptomatic parameters by microbiota of donor’s recipients. Liver (**A**), spleen (**B**), kidneys (**C**), colon (**D**), cecum (**E**), and colon length (**F**) are expressed as ratios of body weight, together with the percentage of weight loss **G**) and disease activity index (**H**). Diets: purified (P), and 25% inclusion of maize NIL with 3-DFs (B), 3-HFs (C), or both 3-DF + 3HF (D). Human fecal donors: H-1, H-2, and H-3. Replicates (*n* = 3–5) are shown for every diet per donor. Statistical differences are represented by (*) *p* ≤ 0.05, and (#) *p* ≤ 0.10.

Flavonoid-rich diets had distinct effects depending on the microbiota of the donor (Figure 3A–H). In H-1 recipients, diet C resulted in reduced cecum weights compared to the P diet. The 3-DF-rich diet increased DAI and showed an increased weight loss pattern compared to the control. Also, diet D (3-DF + 3-HF) prevented weight loss compared to 3-DF alone. In H-2 recipients, none of the diets were effective. As such, diet B reduced colon length, and diet C increased colon weight, weight loss, and DAI. Diet D increased spleen weight and showed a pattern of increasing DAI. However, in this group, 3-HF augmented colon size and DAI, and preserved colon length compared to 3-DF. Moreover, 3-HF alone was not as effective as mixed with 3-DF at preventing weight loss or maintaining colon weight. For H-3 recipients, diet C demonstrated a reduction in DAI compared to the P diet. Furthermore, 3-HFs improved DAI compared to 3-DFs. Representative hematoxylin-and-eosin-stained distal colon sections are shown in Supplementary Figure S6.

### 3.2. Pattern Recognition Receptors (PRR) and Inflammatory Markers

**Figure 4:**
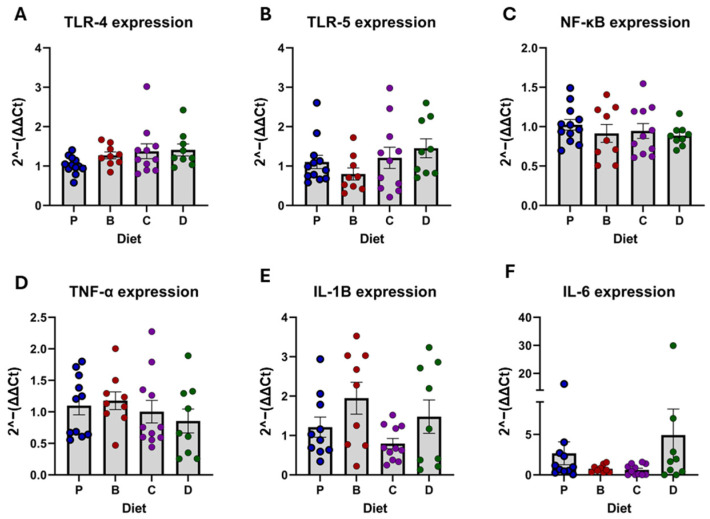
Overall diet effects on PRRs and inflammatory markers. mRNA expression of TLR-4 (**A**), TLR-5 (**B**), NF-κB (**C**), TNF-α (**D**), IL-1β (**E**), and IL-6 (**F**). Diets: purified (P), and 25% inclusion of maize NIL with 3-DFs (B), 3-HFs (C), or both 3-DF + 3HF (D). Replicates (*n* = 9–12) are shown for every diet.

**Figure 5:**
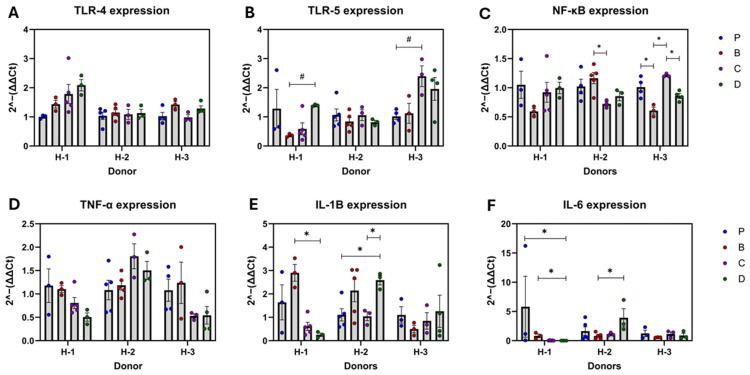
Diet effects on PPRs and inflammatory markers by microbiota of donor’s recipients. mRNA expression of TLR-4 (**A**), TLR-5 (**B**), NF-κB (**C**), TNF-α (**D**), IL-1β (**E**), and IL-6 (**F**). Diets: purified (P), and 25% inclusion of maize NIL with 3-DFs (B), 3-HFs (C), or both 3-DF + 3HF (D). Human fecal donors: H-1, H-2, and H-3. Replicates (*n* = 3–5) are shown for every diet per donor. Statistical differences are represented by (*) *p* ≤ 0.05, and (#) *p* ≤ 0.10.

Overall expression of PRRs and inflammatory markers revealed no significant effects of the flavonoid-rich diets compared to diet P (Figure 4A–F). Although dietary effects on most biomarkers turned significant when focused on each recipient’s donor; toll-like receptor (TLR)-4 and tumor necrotic factor (TNF)-α remained unaffected (Figure 5A–F).

Recipients of donor H-1 showed IL-6 downregulation by diet D compared to P. Moreover, the 3-HF + 3-DF diet reduced IL-1β compared to 3-DF and exhibited a pattern of upregulation of TLR-5. In H-2 recipients, none of the inflammatory markers were reduced by the flavonoid-rich diets compared to the control. However, 3-HF was more effective in maintaining IL-1β expression than in combination with 3-DF, and 3-HF also showed potential for keeping NF-κB stable compared to the 3-DF diet. Furthermore, diet D upregulated IL-1β expression compared to diet P, and increased IL-6 compared to 3-DF alone, showing no synergistic benefit. Nevertheless, treatment effects were different for H-3 recipients, where diet B was effective in reducing NF-κB expression compared to the control. In this scenario, the 3-HF diet was less effective at regulating NF-κB compared to other flavonoid-rich diets.

### 3.3. Tight Junction Proteins and Gut Barrier Integrity

**Figure 6:**
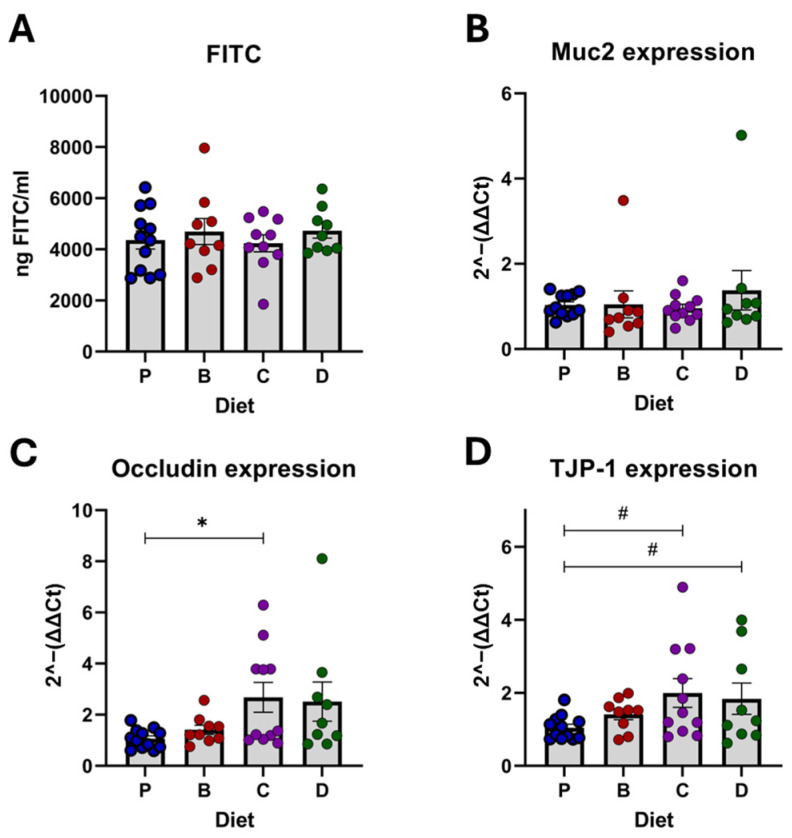
The effect of diet on gut permeability, mucus protection, and tight junction proteins. FITC concentration in serum (**A**), mRNA expression of Muc2 (**B**), occludin (**C**), and TJP-1 (**D**). Diets: purified (P), and 25% inclusion of maize NIL with 3-DFs (B), 3-HFs (C), or both 3-DF + 3HF (D). Replicates (*n* = 9–12) are shown for every diet. Statistical differences are represented by (*) *p* ≤ 0.05, and (#) *p* ≤ 0.10.

**Figure 7:**
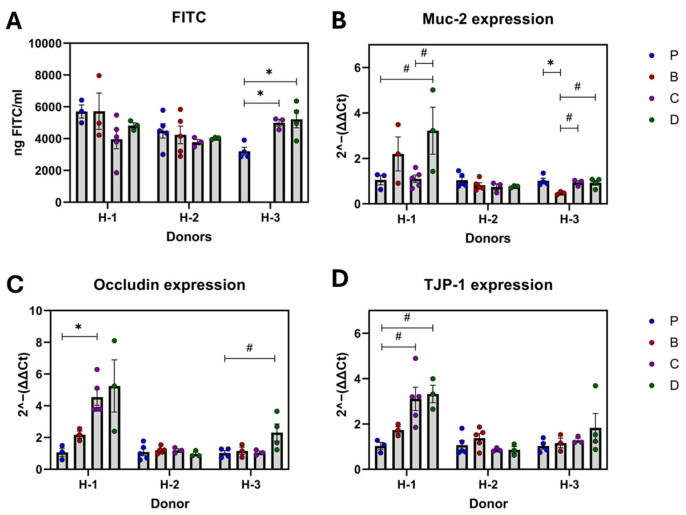
On the effect of diet on gut permeability, mucus protection, and tight junction proteins by the microbiota of each donor’s recipients. FITC serum concentration (**A**), mRNA expression of Muc2 (**B**), occludin (**C**), and TJP-1 (**D**). Diets: purified (P), and 25% inclusion of maize NIL with 3-DFs (B), 3-HFs (C), or both 3-DF + 3HF (D). Human fecal donors: H-1, H-2, and H-3. Replicates (*n* = 3–5) are shown for every diet per donor. Statistical differences are represented by (*) *p* ≤ 0.05, and (#) *p* ≤ 0.10.

Overall results showed gut barrier protection by diet C with upregulated occludin and tight junction protein (TJP)-1 (Figure 6A–D) compared to diet P. However, these effects were attributed to specific donors (Figure 7A–D). In H-1 recipients, diet C increased Occludin and TJP-1, while in H-3 recipients, diet D elevated TJP-1 expression compared to the control. Treatments did not affect Muc2 expression in the overall analysis (Figure 6B). However, 3-HF-rich diets showed upregulation patterns in H-1 recipients while the 3-DF diet downregulated Muc2 in H-3 recipients. No significant effects of diet were observed in H-2 recipients.

In the overall analysis, diets did not show any significant effects on gut membrane permeability, as assessed by FITC, compared to the control (Figure 6A). Surprisingly, diets C and D increased permeability in H-3 recipients, which was inconsistent with the relative gene expression levels of tight junction proteins.

### 3.4. Microbial Diversity and Composition

**Figure 8:**
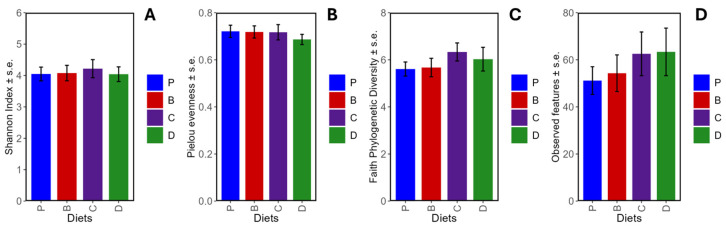
Overall diet effects on α-diversity indexes. Shannon index (**A**), Pielou evenness (**B**), Faith phylogenetic diversity (**C**), and observed features (**D**). Diets: purified (P), and 25% inclusion of maize NIL with 3-DFs (B), 3-HFs (C), or both 3-DF + 3HF (D). Replicates (*n* = 9–12) are shown for every diet.

**Figure 9:**
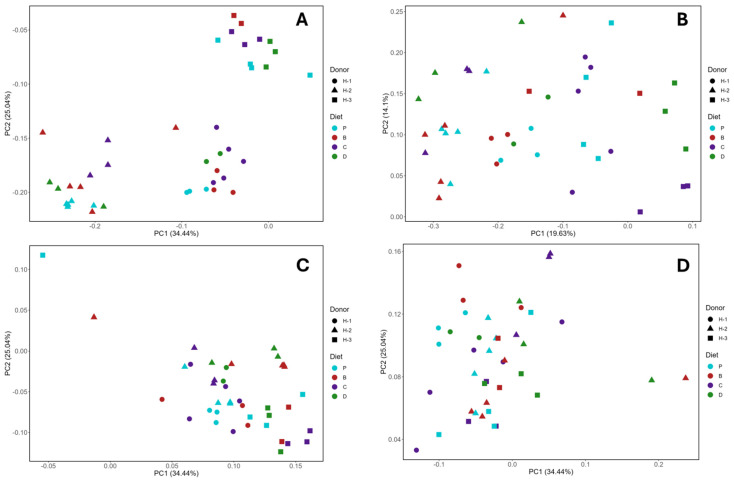
Principal coordinate analysis of β-diversity. Jaccard index (**A**) and Bray–Curtis dissimilarity (**B**), unweighted UniFrac (**C**), and weighted UniFrac (**D**) distances. Diets: purified (P), and 25% inclusion of maize NIL with 3-DFs (B), 3-HFs (C), or both 3-DF + 3HF (D). Human fecal donors: H-1, H-2, and H-3. Replicates (*n* = 9–12) are shown for every diet.

No significant differences in α-diversity indexes (Figure 8A–D) were observed between diets. Furthermore, the β-diversity analysis showed no significant differences in the distance between gut microbial communities across diets (Figure 9A–D). Although clustering by Jaccard index (Figure 9A) indicated unique species variety for each human donor microbiota group. Nevertheless, when relative abundance and phylogenetic relationships were considered (Figure 9B–D), the clustering was not observed. Therefore, the source of microbiota (human donors) was more relevant to diversity than the diet.

**Figure 10:**
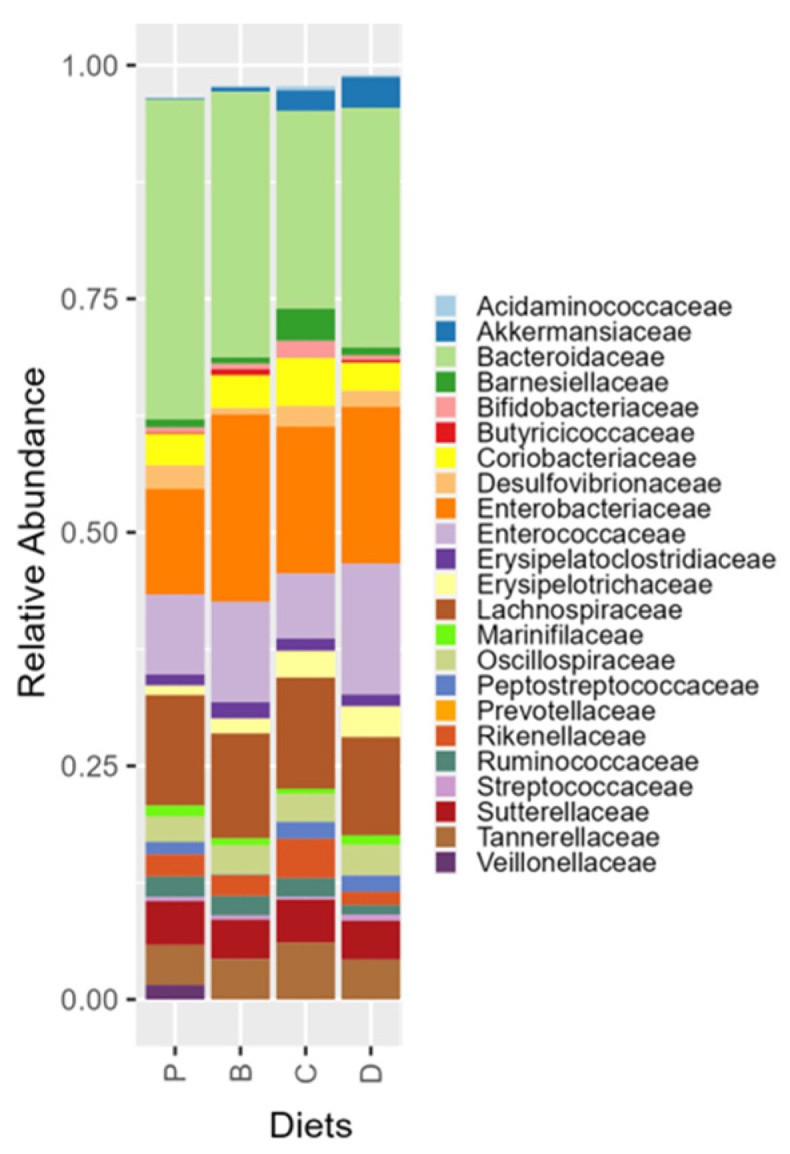
Overall taxonomic diversity plot showing the relative abundance of gut microbiota at the family level per dietary treatment. Taxa higher than 1% relative abundance is plotted. Diets: purified (P), and 25% inclusion of maize NIL with 3-DFs (B), 3-HFs (C), or both 3-DF + 3HF (D).

Compositional analysis revealed diet-based differences at the family level (Figure 10). Compared to the control diet, diet C reduced the relative abundance of *Bacteroidaceae* and *Veillonellaceae*. The microbial composition of recipients on diet B was not significantly different from other diets. The combined activity of the 3-HF + 3-DF diet promoted *Streptococcaceae, Enterococcaceae, and Butyricicoccaceae*, and reduced *Rikenellaceae* compared to the 3-HF diet. Only changes in taxa higher than 1% of the total abundance in the microbiota profile are described, but other significant changes in less abundant taxa can be found in Supplementary Material Table S1.

**Figure 11:**
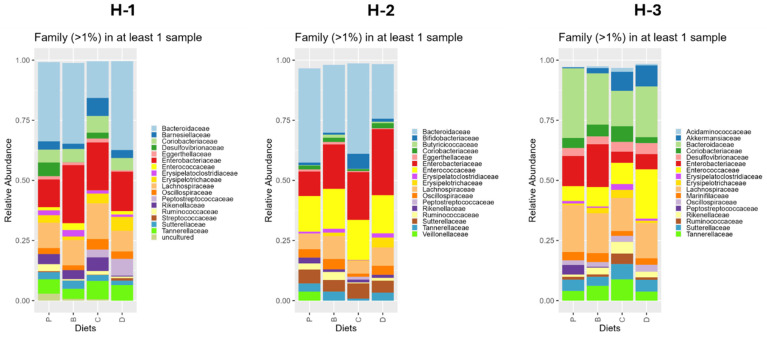
Taxonomic diversity plot showing diet effects in the relative abundance of gut microbiota Families by donor of microbiota. Taxa higher than 1% relative abundance is plotted. H-1, H-2, and H-3 refer to the human fecal donors. Diets: purified (P), and 25% inclusion of maize NIL with 3-DFs (B), 3-HFs (C), or both 3-DF + 3HF (D).

Comparing flavonoid effects vs. the control diet within individual donors, H-1 recipients receiving flavonoid diets had reduced relative abundance of *Ruminococcaceae* (Figure 11), whereas diet C lowered *Bacteroidaceae* and *Enterococcaceae* relative abundance, and diet D diminished *Desulfovibrionaceae* and *Rikenellaceae* abundance. The combinatorial activity of 3-DF + 3-HF favored *Peptostreptococcaceae* compared to diets containing only one of the flavonoids and increased *Erysipelotrichaceae* compared to the 3-DF diet. In H-2 recipients, *Veillonellaceae* was diminished by all flavonoid-rich diets. *Erysipelatoclostridiaceae*, *Butyricicoccaceae*, and *Oscillospiraceae* were reduced by diet C, while *Bifidobacteriaceae* and *Peptostreptococcaceae* were enhanced compared to the control. Likewise, diet D supported *Erysipelatoclostridiaceae* growth. Comparing flavonoid effects, the 3-HF diet was more effective than 3-DF in increasing the relative abundance of *Bifidobacteriaceae*. The combined activity of 3-DF + 3-HF increased *Erysipelatoclostridiaceae* and reduced *Bacteroidaceae* and *Bifidobacteriaceae* compared to 3-HF. In H-3 recipients, diet P mice exhibited the highest abundance of *Peptostreptococcaceae* compared to all the other diets. Diet B decreased the abundance of *Erysipelatoclostridiaceae* and *Ruminococcaceae*, while anthocyanin-rich diets enhanced *Akkermansiaceae*. When contrasting the flavonoid diet activity, 3-HFs depleted *Enterobacteriaceae* and promoted *Akkermansiaceae* and *Ruminococcaceae* compared to 3-DFs. Moreover, the presence of 3-HF reduced *Peptostreptococcaceae* compared to 3-DF. Conversely, the mixture of 3-DF + 3-HF increased *Enterococcaceae* compared to 3-DF and 3-HF alone, diminished *Ruminococcaceae* compared to 3-HF, and increased *Enterobacteriaceae* and *Akkermansiaceae* compared to 3-DF.

### 3.5. Microbial Metabolic Products: SCFA and Bile Acids

**Figure 12:**
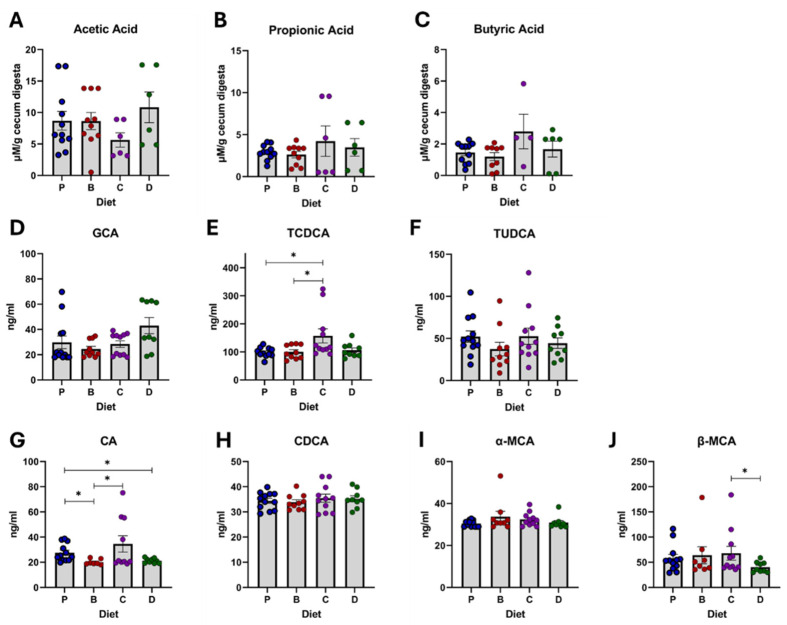
Overall diet effect on gut metabolites: short-chain fatty acids and primary bile acids. SCFAs: acetate (**A**), propionate (**B**), butyrate (**C**), and bile acids: glycocholic acid, GCA (**D**), taurochenodeoxycholic acid, TCDCA (**E**), tauroursodeoxycholic acid, TUDCA (**F**), cholic acid, CA (**G**), chenodeoxycholic acid, CDCA (**H**), α-murocholic acid, α-MCA (**I**) and β-murocholic acid, β-MCA (**J**). Diets: purified (P), and 25% inclusion of maize NIL with 3-DFs (B), 3-HFs (C), or both 3-DF + 3HF (D). Replicates (*n* = 9–12) are shown for every diet. Statistical differences are represented by (*) *p* ≤ 0.05.

**Figure 13:**
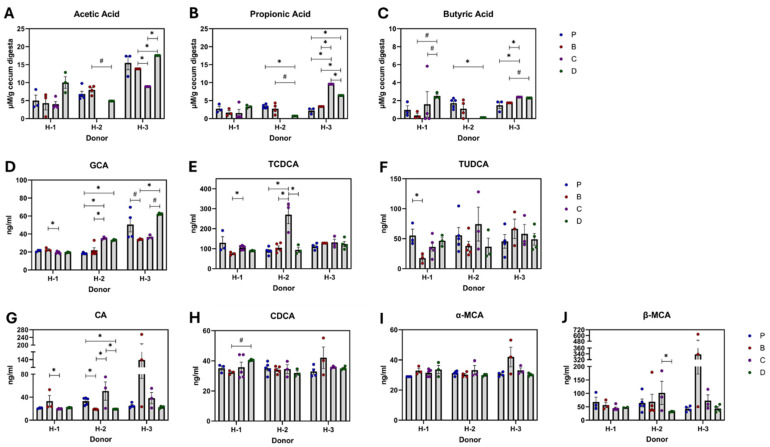
Dietary effect on gut metabolites: short-chain fatty acids and primary bile acids by microbiota of donor’s recipients. SCFAs: acetate (**A**), propionate (**B**), butyrate (**C**), and bile acids: glycocholic acid, GCA (**D**), taurochenodeoxycholic acid, TCDCA (**E**), tauroursodeoxycholic, TUDCA (**F**), cholic acid, CA (**G**), chenodeoxycholic acid, CDCA (**H**), α-murocholic acid, α-MCA (**I**) and β-murocholic acid, β-MCA (**J**). Diets: purified (P), and 25% inclusion of maize NIL with 3-DFs (B), 3-HFs (C), or both 3-DF + 3HF (D). Human fecal donors: H-1, H-2, and H-3. Replicates (*n* = 3–5) are shown for every diet per donor. Statistical differences are represented by (*) *p* ≤ 0.05, and (#) *p* ≤ 0.10.

Large variability was found when donors were pooled to compare diet effects on the SCFA concentration, resulting in non-significant effects (Figure 12A–J). However, the recipient’s microbiota donor approach allowed observation of multiple trends (Figure 13A–J). In H-1 recipients, diets did not significantly change butyrate production, however, 3-DF + 3-HF together showed more potential to maintain butyrate concentration than diets with only 3-DF or 3-HF. Conversely, in H-2 recipients, diet D depleted the production of propionate and butyrate compared to the control. The effects for the H-3 recipients were more diverse. Compared to the control, only diet C improved propionate and butyrate. Similarly, 3-HF increased propionate and butyrate compared to 3-DF. Diet D increased propionic acid levels compared to the control.

Several primary bile acids were quantified, but only a few were altered by diets. When compared to the purified diet, the presence of 3-DFs (diets B and D) reduced the CA level, but the 3-HF diet (diet C) maintained it. Conversely, diet C elevated TCDCA compared to the control and diet B. Overall, TCDCA results were justified by the patterns of H-2 and H-1 recipients. In H-1 recipients, phlobaphene diets reduced TUDCA compared to the control. Reductions in CA and GCA and restoration of TCDCA by 3-HF compared to 3-DF suggest distinct modulatory roles of phlobaphenes and anthocyanins. In H-2 recipients, diet C increased GCA and TCDCA compared to the control and elevated CA, GCA, and TCDCA compared to 3-DF. The combination of 3-DF + 3-HF was not as effective as 3-HF alone in increasing CA or TCDCA. In contrast, in H-3 recipients, the 3-DF + 3-HF combination maintained GCA levels compared to the single compounds.

### 3.6. Associations of Gut Bacteria Composition with Inflammatory Markers, Tight Junction Proteins, Mucus Production, and Gut Metabolites

Positive (+) and negative (−) correlations were analyzed to identify associations between the relative abundance of bacteria and the gene expression of the evaluated biomarkers (Supplementary Figure S1). Moderate and high correlations (r > 0.40) were observed between TLR-4 expression and *Barnesiellaceae* (+) and *Veillonellaceae* (−), while TLR-5 was correlated with *Eggerthellaceae* (−), *Enterobacteriaceae* (−), *Akkermansiaceae* (+), *Acidaminococcaceae* (+), and *Marinifilaceae* (+). For inflammatory cytokines, IL-6 was associated with *Barnesiellaceae* (−) and *Rikenellaceae* (−), IL-1β with *Enterobacteriaceae* (+) and *Desulfovibrionaceae* (−), and TNF-α with *Enterobacteriaceae* (+), *Desulfovibrionaceae* (−), and *Rikenellaceae* (−). NF-κB expression was correlated to *Ruminococcaceae* (+) and *Butyricicoccaceae* (+). Biomarkers directly associated with the gut barrier integrity and muc2 expression were not highly correlated to any bacteria taxa; however, occludin was associated with *Barnesiellaceae* (+) and *Eubacteriaceae* (−), and TJP-1 was found to be associated with *Barnesiellaceae* (+) and *Enterococcaceae* (−).

Following their similarities in the association with the microbiota relative abundance, the biomarkers were hierarchically clustered in three groups including markers related to inflammation (IL-6, IL-1β, and TNF-α), gut barrier integrity (Muc2, occludin, and TJP-1) and TLRs with the inclusion of NF-κB (TLR-4, and NF-κB, and TLR-5). Certain bacteria influenced the expression of more than one marker in each of these groups, and we interpreted that as a potential involvement in the activity of those genes. *Enterobacteriaceae* was associated more with the pro-inflammatory activity, *Desulfovibrionaceae* and *Rikenellaceae* with the anti-inflammatory activity, and *Barnesiellaceae* with the protection of gut barrier integrity. However, only *Enterobacteriaceae* and *Rikenellaceae* were present in all three donor-recipient pairs with relative abundances exceeding 1%. In contrast, *Desulfovibrionaceae* surpassed the 1% threshold only in recipients H-1 and H-3, while *Barnesiellaceae* exceeded 1% only in H-1 recipients. Overall, *Enterobacteriaceae* was the most abundant taxon and showed a strong correlation with several biomarkers.

Only a few bacteria families demonstrated a moderate to high correlation with the three SCFAs (Supplementary Figure S2). *Akkermansiaceae and Acidaminococcaceae* showed positive correlations, and *Eggerthellaceae* demonstrated negative correlations with all the SCFAs. *Enterobacteriaceae* had the strongest negative correlation (r > 0.6) with propionate and butyrate, but a mild negative correlation (r < 0.4) with acetate. Furthermore, acetate content was correlated with *Marinifilaceae (+) and Barnesiellaceae (−).* Propionate was correlated with *Ruminococcaceae* (+), *Enterobacteriaceae* (−), *Anaerovoracaceae* (+), *Eubacteriaceae* (+), and *[Eubacterium]_coprostanoligenes_group* (+). Finally, butyrate was strongly associated with *Enterobacteriaceae* (−). Of all the families mentioned above, only *Enterobacteriaceae* and *Ruminococcaceae* were part of every recipient’s donor group in more than 1% of the profile composition, with *Enterobacteriaceae* being the most abundant.

Bile acids were associated with most of the taxa mentioned (Supplementary Figure S3). GCA was positively correlated (r > 0.6) with *Akkermansiaceae*, *Acidaminococcaceae*, and *Marinifilaceae*, and moderately correlated (r > 0.4) with *Desulfovibrionaceae* and *Clostridiaceae*. Also, moderate negative correlations were found with *Bifidobacteriaceae*, *Veillonellaceae*, and *Eggerthellaceae*. Conversely, CA, which is the unconjugated form of GCA, only showed weak correlations. TCDCA showed a moderate positive correlation with *Akkermansiaceae*. Furthermore, TUDCA only had a moderate negative correlation with *Enterobacteriaceae*. Neither α-MCA nor β-MCA correlated higher than 0.4 with any of the taxa.

### 3.7. Correlations Among and Within Inflammatory Markers, Disease Activity, and Gut Metabolites

**Figure 14:**
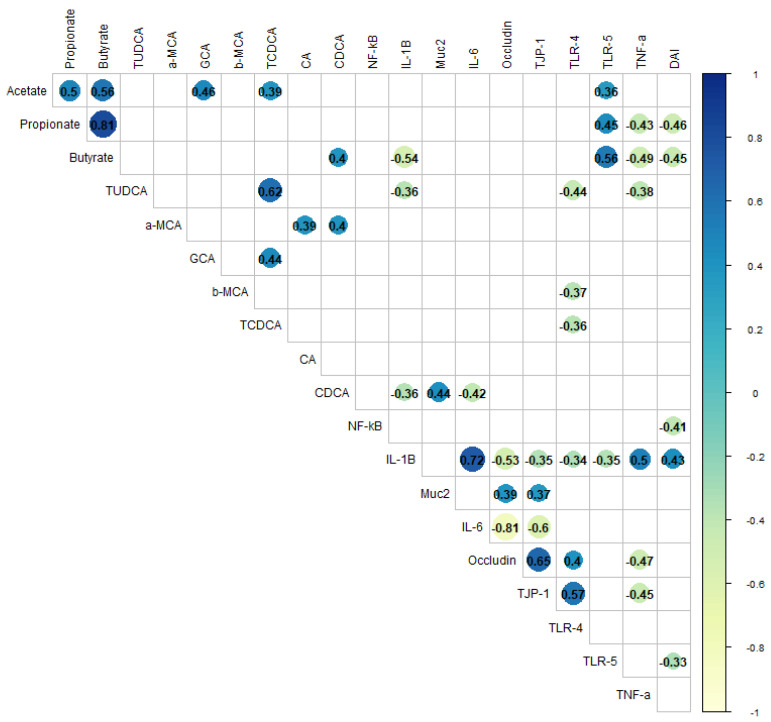
Correlation heatmap between biomarkers and gut metabolites. Darker colors represent higher positive correlations, while lighter represent highly negative correlations. Only significant *p* < 0.05 correlations are shown. Pattern recognition receptors: toll-like receptor-4, TLR-4, toll-like receptor-5, TLR-5. Inflammatory markers: interleukin-1β, IL-1β, interleukin 6, IL-6, tumor necrotic factor- α, TNF-α. Tight junction proteins: occludin and tight junction protein 1, TJP-1. Mucin 2: Muc2. Disease activity index: DAI. Bile acids: glycocholic acid, GCA, taurochenodeoxycholic acid, TCDCA, tauroursodeoxycholic, TUDCA, cholic acid, CA, chenodeoxycholic acid, CDCA, α-murocholic acid, α-MCA, and β-murocholic acid, β-MCA.

Correlations were analyzed to find associations between gut metabolites and colitis biomarkers (Figure 14). Only three inflammatory markers were associated with SCFA production. TLR-5 was positively correlated with all SCFAs. TNF-α was negatively correlated with propionic and butyric acid, while IL-1β was negatively associated only with butyric acid. This suggests the anti-inflammatory potential of propionic and butyric acid. Regarding the DAI, negative correlations were observed with NF-κB, TLR-5, propionic, and butyric acid, but positive correlations were found with IL-1β. This reinforces the potential benefits of propionic and butyric acid and targets IL-1β as the most associated marker with disease symptoms. TUDCA showed mild negative correlations with TLR-4, IL-1β, and TNF-α; β-MCA and TCDCA only with TLR-4. CDCA was also negatively associated with IL-1β and IL-6, but positively with Muc2.

When observing associations within the biomarkers, the strongest positive associations were found between IL-1β and IL-6 (r = 0.72), occludin and TJP-1 (r = 0.65), and TLR-4 and TJP-1 (r = 0.57), In contrast, occludin showed the highest negative correlations against IL-6 (r = −0.81) and IL-1β (r = −0.53), which supports the impairment of the gut barrier integrity promoted by expression of pro-inflammatory cytokines. SCFAs were positively correlated with each other. Propionic and butyric acid had the highest correlation (r = 0.81). TUCDA and TCDCA had the highest correlation (r = 0.62) within the bile acids.

Overall, IL-1β showed positive correlations with other cytokines and disease activity and negative correlations with TLRs and tight junction protein expression.

## 4. Discussion

Global disease statistics suggest the highest incidence and prevalence of ulcerative colitis (UC) in the United States [22]. Even when pathogenesis is not completely clear, disease development involves gut dysbiosis and dietary habits. Bacterial dysbiosis represents an imbalance in the composition and function of the microbiota, triggered by multiple environmental and genetic factors, which lead to mucosal immune dysfunction [23]. Moreover, dietary choices can ameliorate (high fiber, fruits, and vegetables) or aggravate (high-fat, high-sugar, or processed food) the risk and progression of the disease [24]. In alignment, dietary bioactive compounds like flavonoids can be an alternative for disease alleviation. Mice model studies with NILs have shown the potential benefits of phlobaphenes and anthocyanins within a corn matrix [10,11]. This study using a humanized mice colitis model and flavonoid-rich corn NILs showed gut barrier protective effects and distinct microbiota compositions compared to the purified diet but did not demonstrate significant anti-inflammatory properties. Responses to the diets exhibited high variability across donors. This may be due to the inherent variability in microbiota colonization and the host-microbiota interactions. However, when results were analyzed using recipients of a single donor, variability was narrowed and dietary effects were significant, following distinct patterns within each donor group.

In H-1 recipients, anthocyanin-rich diets demonstrated anti-colitic activity compared to the control. The beneficial activity was consistent with the effects of phlobaphene- and anthocyanin-rich NILs of corn in previous studies involving mice-native microbiota [10]. Both diets C and D upregulated tight junction proteins, but diet D also exhibited anti-inflammatory properties (IL-6 reduction) and stimulated mucus production. We suggest that the benefits of diet D may be related to an increase in the relative abundance of *Peptostreptococcaceae* and a reduction in *Desulfovibrionaceae*. An increased abundance of *Peptostreptococcaceae* is considered a microbiota signature for the alleviation of Crohn’s disease [25], an IBD condition, while *Desulfovibrionaceae*, a group of sulfate-reducing bacteria, are more abundant in IBD patients [26]. *Desulfovibrionaceae* can produce endotoxins that disrupt the mucus barrier or promote intestinal permeability through dysbiosis and have been reported to negatively associate with TJP-1 expression [27,28], indicating that lower abundance favors gut barrier health. Conversely, other studies suggest that certain species of *Desulfovibrionaceae* are reduced in IBD because they only degrade mucus under acidic conditions, which are not typical in this disease [29]. Additionally, diets containing 3-DF reduced *Desulfovibrionaceae* abundance, suggesting a potential modulatory role. However, the anti-colitic effects observed with diet D were not observed with diet B, possibly associated with the higher level of *Enterobacteriaceae*, which moderately correlated with pro-inflammatory markers (IL-1β and TNF-α). *Enterobacteriaceae*, a facultative anaerobe typical in IBD, proliferates when inflammatory conditions limit β-oxidation in intestinal cells creating a favorable environment [30]. Anthocyanins in the C and D diets may promote conditions that limit *Enterobacteriaceae* dominance. In addition, the improvement of tight junctions by diet C could be associated with a reduction in *Enterococcaceae*, which was negatively correlated with TJP-1. *Enterococcaceae*, particularly *Enterococcus faecalis*, is another taxon characteristic of IBD, which is identified as the only commensal bacteria used for inducing IBD in mice models [31]. This saccharolytic group of bacteria is suspected to participate in a cross-feeding dynamic in mucin degradation [32], thereby disturbing the gut barrier. Collectively, results obtained from H-1 recipients suggest a barrier protection role of 3-HFs and anti-inflammatory potential when combined with 3-DFs. However, claiming synergistic activity requires further study and a better understanding of the interactions with the gut microbiota composition.

Opposite to the patterns observed in H-1 recipients, flavonoid-rich diets did not alleviate inflammation or improve gut barrier function in H-2 recipients compared to the control. In general, diet B affected the least number of parameters evaluated, only reducing colon length and BA levels. Diet C aggravated UC symptoms, while diet D exacerbated inflammation and reduced SCFAs. The promotion of *Erysipelatoclostridiaceae* by the D diet was consistent with this taxa’s pathogenic potential and association with IBD in DSS mice models [33], even though certain polyphenol-rich grain extracts demonstrated potential in reducing this taxon [34]. Additionally, *Veillonellaceae* abundance is primarily associated with UC [35] but its presence is limited by disease severity [36], which may explain the reduction caused by the D diet where symptoms were more intense. Also, a reduction in *Eggerthellaceae* by diet D might be associated with inflammation, as members of this taxon possess mucus degradation genes and produce anti-inflammatory metabolites [37]. Furthermore, a reduction in *Veillonellaceae* and *Eggerthellaceae* was associated with an increase in GCA, which is common in IBD patients [38,39]. We also suggest contrasting effects of phlobaphenes and anthocyanins on *Butyricicoccaceae*, given that diet B increased this taxon, diet C reduced it, and diet D balanced both effects compared to the control. When *Butyricicoccaceae* levels were low, disease symptoms and inflammation worsened, which is consistent with a reduction in *Butyricicoccaceae* in IBD patients and the butyrate-producing attributes of this taxon [40]. Even when our results did not show strong correlations between the mentioned taxa and the SCFA production, the negative correlations between SCFAs and inflammation parameters (IL-1β and DAI), suggest a modulatory role of the gut microbiota through their metabolites. Both butyrate and propionate have multiple mechanisms of action to reduce inflammation in the gut. We speculate that changes in the SCFA profile would be more dependent on the overall microbiota composition, even when differences in abundance were not statistically significant between diets. We also suggest that the *Enterobacteriaceae* increment by diet D can be associated with propionate and butyrate reduction, which was supported by the correlations. Lastly, even when associations with bacteria and metabolites might explain the activity of diets regarding inflammation, the reason for the lack of anti-inflammatory effects in this recipient’s group remains uncertain. We hypothesize that the microbial composition at colonization and consequent bacterial metabolism might play a significant role in the anti-colitic effects of phlobaphenes and anthocyanins.

In H-3 recipients, the presence of *Akkermansiaceae* was potentially involved in the effects of the flavonoid-rich diets. The anthocyanin-rich diets, C and D, improved disease activity and tight junctions, respectively, along with increased production of SCFA compared to diet P. *Akkermansiaceae* was enriched in the C and D diets, exclusively *Akkermansia muciniphila*. This bacteria is a mucus-degrading species that blooms in gut environments after microbiota depletion, as demonstrated by the colonization of pseudo-germ-free mice [41]. The role of *Akkermansiaceae* in colitis is contradictory, as it can be pro- or anti-inflammatory [42]. *A. muciniphila* extracellular products, such as proteins and SCFAs, can contribute to enhancing gut barrier integrity. For example, the pilus-associated signaling (PAS) proteins in the cell membrane of *A. muciniphila* stimulate TLR-2 [43], which upregulates tight junction proteins and IL-10 expression [44]. Given the increase in *A. muciniphila,* we expected an associated anti-inflammatory response. However, the IL-10 -/- condition of our model is a potential limitation for the potential *Akkermansiaceae* full restorative effects, especially over inflammation. Previous research has demonstrated associations between *A. muciniphila* growth, lipocalin-2, and weight loss in DSS-induced colitis [42], and limited anti-inflammatory activity in IL-10 -/- models. The interactions with other bacteria in the gut could explain the pro- or anti-inflammatory activity of *Akkermansiaceae* [45]. The abundance of *Enterococcocea* (even when not statistically significant) and their association with TJP-1 reduction may be associated with the alleviation of inflammation and gut permeability. Anthocyanin-rich diets have increased *Akkermansiaceae* in diverse colitis or inflammatory mice models [46,47], similar to what we observed in this study. In addition, our results reveal a moderate association between *Akkermansiaceae* and TLR-5. Paradoxically, *A. muciniphila* lacks flagellin among the pili proteins [48] to directly influence this receptor. Nevertheless, TLR-5 can be regulated by mechanisms other than flagellin induction, like IFN-γ, which downregulates TLR-5 after colitis induction in DSS models [49]. Finally, the higher levels of propionate and butyrate in anthocyanin-rich diets are moderately correlated with *Akkermansiaceae*, as a potential consequence of its enrichment [43]. Propionate could contribute to alleviating inflammation through mechanisms like Treg cell upregulation and proliferation [50].

Previous studies with DSS-induced colitis mice models demonstrated that the presence and interaction with the gut microbiota are essential for 3-DFs and 3-HFs to exert anti-inflammatory effects [10]. Our results showed that these effects depend on the microbial composition, involving the uniqueness of the microbiota of each human donor and the effectiveness of colonization in the mice. Nevertheless, when we only focus on the differences between 3-DF and 3-HF diets within the same food matrix, anthocyanins tend to be more beneficial than phlobaphenes. Anthocyanins demonstrated a greater capacity to restore gut barrier integrity (H-1, H-3), alleviate disease symptoms (H-1 and H-3), reduce nuclear factors of inflammation (H-2), induce bile acid production, and increase SCFA concentration (H-1 and H-3), whereas phlobaphenes only improved a few gut metabolites. Conversely, the combined effects of 3-DF and 3-HF were heterogeneous among recipient donors. Compared to the anthocyanin diet, the 3-DF + 3-HF combination was more effective in restoring gut barrier integrity (H-1, H-3) and stimulating butyrate production (H-1), but failed to reduce inflammatory markers (H-2) and nuclear factors of inflammation (H-3) or maintain BA levels (H-2) and propionate production (H-3). This is consistent with the large amount of research on anthocyanin’s benefits through gut metabolism. Gut microbial enzymes like β-glucosidase, α-galactosidase, and α-rhamnosidase allow the metabolism of anthocyanins into phenolic acids and multiple metabolic by-products, which modulate the gut environment to exert health benefits [51]. Conversely, studies on phlobaphenes are more limited [11]. We hypothesize that their complexity given by their degree of polymerization [52] limits their accessibility, only allowing selective bacteria to metabolize them, as is proposed in simulations with complex polyphenols [53]. However, 3-DF and 3-HF are utilized differently by the gut microbiota, and their potential synergy could be convenient under specific gut conditions.

One of the limitations of our study is the small sample size per donor and the translation of the results to population level or humans. The sample size is important to depict bacteria dynamics and networking, reducing individual sample variability, and thus allowing us to identify more relevant trends. More variables still need to be studied like the effects of diet, physiology, sex, and age of the donors besides the multiplicity of bacterial community compositions and associated health conditions that can be found in humans. However, our study shows the impact that different microbial communities have on the activity of flavonoids, even when bacteria come from healthy individuals. Future directions of our study include a larger variety of donors and networking analysis to identify potential bacteria interactions behind the effects on gut health across donors.

## 5. Conclusions

In conclusion, our findings demonstrate that the effects of 3-DF and 3-HF on gut inflammation and gut barrier protection depend on the composition of human-associated microbiota. Overall, anthocyanin-rich diets exhibited greater versatility than phlobaphenes in their capacity to improve colitis-associated conditions, though their potential synergism requires further study.

## Figures and Tables

**Table 1:** Composition of experimental diets.

Ingredients	Diet P	Diet B	Diet C	Diet D
	TD.97184	TD.220428	TD.220429	TD.220430
	g/kg	g/kg	g/kg	g/kg
Casein	200.0	166.1	162.156	158.582
L-Cystine	3.0	3.0	3.0	3.0
Corn Starch	392.234	192.147	189.456	202.679
Maltodextrin	132.0	132.0	132.0	132.0
Sucrose	100.0	100.0	100.0	100.0
Soybean Oil	70.0	58.591	58.604	56.643
Cellulose	50.0	17.0	23.0	15.0
Mineral Mix, AIN-93G-MX (94046)	35.0	35.0	35.0	35.0
Vitamin Mix, AIN-93-VX (94047)	15.0	15.0	15.0	15.0
Choline Bitartrate	2.75	2.75	2.75	2.75
Vitamin K1, phylloquinone	0.002	0.002	0.002	0.002
TBHQ, antioxidant	0.014	0.014	0.014	0.014
Maize NIL B		278.4		
Maize NIL C			279.0	
Maize NIL D				279.3

**Table 2:** rtPCR primers of colonic inflammatory markers.

Gene	Primers
Il1b	Forward: 5′-GCCCATCCTCTGTGACTCAT-3′ Reverse: 5′-AGGCCACAGGTATTTTGTCG-3′
Il6	Forward: 5′-AGTTGCCTTCTTGGGACTGA-3′ Reverse: 5′-CAGAATTGCCATTGCACAAC-3′
Tnfa	Forward: 5′-CAAAATTCGAGTGACAAGCCTG-3′ Reverse: 5′-GAGATCCATGCCGTTGGC-3′
Nfkb	Forward: 5′-ACCTTTGCTGGAAACACACC-3′ Reverse: 5′-ATGGCCTCGGAAGTTTCTTT-3′
Ocln	Forward: 5′-ACGGACCCTGACCACTATGA-3′ Reverse: 5′-TCAGCAGCAGCCATGTACTC-3′
Muc2	Forward: 5′-GCTGACGAGTGGTTGGTGAATG-3′ Reverse: 5′-GATGAGGTGGCAGACAGGAGAC-3′
Tjp1	Forward: 5′-AGGACACCAAAGCATGTGAG-3′ Reverse: 5′-GGCATTCCTGCTGGTTACA-3′
Tlr4	Forward: 5′-AGTGCCCCGCTTTCACCTCT-3′ Reverse: 5′-TCCGGCTCTTGTGGAAGCCT-3′
Tlr5	Forward: 5′-GCTTGGAACATATGCCAGACACA-3′ Reverse: 5′-AAAGGCTATCCTGCCGTCTGAA-3′
Actb	Forward: 5′-AGCCATGTACGTAGCCATCC-3′ Reverse: 5′-CTCTCAGCTGTGGTGGTGAA-3′

## Data Availability

The original contributions presented in the study are publicly available. Sequences were deposited in the NCBI sequence read archive (SRA) database under Bioproject PRJNA1185774.

## Supplementary Materials

The following supporting information can be downloaded at: https://www.mdpi.com/article/10.3390/nu16234232/s1, Figure S1: Correlation hierarchical heatmap of bacteria family abundance by biomarkers; Figure S2: Correlation hierarchical heatmap of bacteria family abundance by short chain fatty acids; Figure S3: Correlation hierarchical heatmap of bacteria family abundance by primary bile acids; Figure S4: Body weight loss / gain (Average percentage ± SEM) per treatment (n=9-12) during experimental period; Figure S5: Food intake (Average ± CI) per treatment (n=9-12) during experimental period; Figure S6: Representative histological photomicrographs of hematoxylin and eosin (H&E)-stained paraffin longitudinal section of distal colon tissues; Table S1: ANCOM-BC analysis for dietary effects on bacteria family relative abundance; Method S1: Conditions of gas chromatography for SCFA analysis.
