# Supplementary material for: Composition of Human-Associated Gut Microbiota Determines 3-DF and 3-HF Anti-Colitic Activity in IL-10 -/- Mice
Source: Nutrients. 2024 Dec 7;16(23):4232. doi: 10.3390/nu16234232 (PMC11644234; doi:10.3390/nu16234232)
Supplement: Supplementary file 1 [file nutrients-16-04232-s001.zip › nutrients-3356949-supplementary.pdf]

Supplementary Material

Table S1. ANCOM-BC analysis for dietary effects on Bacteria Family Relative Abundance. q-values ≤0.05 are significant.

Overall by P	dietB	dietC	dietD	Family
64fca3239617dac24f5f4c4093ba5bcb	0.9989881	0.3338319	0.4325218	Gastranaerophilales
683e591a876d34cb23e8a68e9fdb8f1a	0.9989881	0.9649944	0.9551077	Coriobacteriales_Incertae_Sedis
4130f21ae0c17450e39a56b46c19737b	0.9989881	0.8228571	0.1017068	Eggerthellaceae
92b4c4b1ddd22276bfdc429a24b3c240	0.9989881	0.8228571	0.9956786	Coriobacteriaceae
f60ff9fcfe17e964a8f857677cd5554a	0.4351364	0.3179652	0.0728448	Atopobiaceae
62df0bf2a96a354cb6311ca343ab768a	0.9989881	0.8228571	0.9929192	Bifidobacteriaceae
49450d9736f3e6d2253eea2117bada10	0.9989881	0.3338319	0.4325218	Akkermansiaceae
82016bad4d8e52fd00ca49f66df848e5	0.9989881	0.9588538	0.9551077	Desulfovibrionaceae
a131a5f05f0233f0f1b6539ee20910ac	0.9989881	0.8228571	0.9551077	Enterobacteriaceae
26aa8aa789a10616d1f469f61638b565	0.9989881	0.5516992	0.9956786	Sutterellaceae
2b466ca5dd30c6518367775d70903ca5	0.9989881	0.5129008	0.9956786	Erysipelatoclostridiaceae
a2d22d7ac96caf193608a148f07920e7	0.9989881	0.0811229	0.6223429	Erysipelotrichaceae
fd63bc6df4da85bdcb38ace3fa4255ff	0.9989881	0.521938	0.4325218	Streptococcaceae
857d9fcb43282b31d88574dc2160b7e6	0.9989881	0.1062101	0.8827159	Enterococcaceae
ccdaecaebacabdf130765573dbb8d2fc	0.9989881	0.7854776	0.9164285	Acidaminococcaceae
b9c6b2168e9b371e0746e2d064cc3bf6	0.2724076	0.0269715	0.0728448	Veillonellaceae
650ab48868fbc4632633a40d8798bdef	0.9989881	0.0269715	0.4325218	Bacteroidaceae
055eac8ba0268dce24092a805d208e9d	0.9989881	0.8228571	0.9551077	Marinifilaceae
c21c4ed964690297593f43867af9a430	0.9989881	0.644706	0.9956786	Barnesiellaceae
d3e105121385c0368f6ac84df9284d13	0.9989881	0.8228571	0.9551077	Tannerellaceae
13577116fdb95c63a3ebd019575c85ff	0.9989881	0.2642463	0.9956786	Rikenellaceae
668a510f30c8545ea672acbf3e20ee1a	0.9997894	0.521938	0.9551077	Anaerovoracaceae
8a9a71b529e55e98b0b76a1ec283d9b7	0.9989881	0.521938	0.6428114	Anaerofustaceae
bf7ad91e744ca9ef821623e115c33846	0.9989881	0.521938	0.9956786	Eubacteriaceae
442c301329744bdb4e54744745b02836	0.9989881	0.8228571	0.9956786	Peptostreptococcaceae
47d86d4a1a6bf75977fe65b65660fd91	0.9989881	0.8228571	0.9551077	Clostridiaceae
c1858e9b447d3109a5da8aec4eb4c153	0.9989881	0.7358489	0.9956786	f_Christensenellaceae
c24e90c57a551a138dd36a3663193921	0.9989881	0.8228571	0.1099082	uncultured
0205de5ae2a84ec03e4e790220f25fea	0.9989881	0.1705828	0.4325218	Butyricicoccaceae
50c1ae203d1f1092d7a47aeda64518e5	0.9997894	0.0269715	0.9956786	Ethanoligenenaceae
e425cd6ab853e65dfceba9433e7faf1e	0.9989881	0.2035423	0.0728448	Ruminococcaceae
f6eb75bbd3ec802e7a412e620d2b3601	0.9989881	0.8228571	0.9724612	[Eubacterium]_coprostanoligenes_group
80d8fc2fb93da3c2a7f31aa48cb5b30d	0.9989881	0.7358489	0.8827159	Oscillospiraceae
c4441181d2378d96e307b1f3e31cbfb6	0.9989881	0.8228571	0.9551077	Lachnospiraceae
Overall by B	dietP	dietC	dietD	Family
64fca3239617dac24f5f4c4093ba5bcb	0.9989881	0.7055092	0.5655701	Gastranaerophilales
683e591a876d34cb23e8a68e9fdb8f1a	0.9989881	0.7681701	0.8658076	Coriobacteriales_Incertae_Sedis

4130f21ae0c17450e39a56b46c19737b	0.9989881	0.9380577	0.8658076	Eggerthellaceae
92b4c4b1ddd22276bfdc429a24b3c240	0.9989881	0.9380577	0.8658076	Coriobacteriaceae
f60ff9fcfe17e964a8f857677cd5554a	0.4351364	0.7681701	0.8658076	Atopobiaceae
62df0bf2a96a354cb6311ca343ab768a	0.9989881	0.720447	0.8658076	Bifidobacteriaceae
49450d9736f3e6d2253eea2117bada10	0.9989881	0.5119505	0.8658076	Akkermansiaceae
82016bad4d8e52fd00ca49f66df848e5	0.9989881	0.4866336	0.8658076	Desulfovibrionaceae
a131a5f05f0233f0f1b6539ee20910ac	0.9989881	0.5119505	0.8658076	Enterobacteriaceae
26aa8aa789a10616d1f469f61638b565	0.9989881	0.7055092	0.8658076	Sutterellaceae
2b466ca5dd30c6518367775d70903ca5	0.9989881	0.4866336	0.8658076	Erysipelatoclostridiaceae
a2d22d7ac96caf193608a148f07920e7	0.9989881	0.346483	0.8658076	Erysipelotrichaceae
fd63bc6df4da85bdcb38ace3fa4255ff	0.9989881	0.4866336	0.8658076	Streptococcaceae
857d9fcb43282b31d88574dc2160b7e6	0.9989881	0.1613955	0.8658076	Enterococcaceae
ccdaecaebacabdf130765573dbb8d2fc	0.9989881	0.7681701	0.8658076	Acidaminococcaceae
b9c6b2168e9b371e0746e2d064cc3bf6	0.2724076	0.7055092	0.9705463	Veillonellaceae
650ab48868fbc4632633a40d8798bdef	0.9989881	0.4866336	0.9173698	Bacteroidaceae
055eac8ba0268dce24092a805d208e9d	0.9989881	0.9380577	0.8658076	Marinifilaceae
c21c4ed964690297593f43867af9a430	0.9989881	0.7055092	0.9210529	Barnesiellaceae
d3e105121385c0368f6ac84df9284d13	0.9989881	0.8924151	0.8658076	Tannerellaceae
13577116fdb95c63a3ebd019575c85ff	0.9989881	0.346483	0.8658076	Rikenellaceae
668a510f30c8545ea672acbf3e20ee1a	0.9997894	0.6823936	0.8658076	Anaerovoracaceae
8a9a71b529e55e98b0b76a1ec283d9b7	0.9989881	0.5119505	0.8658076	Anaerofustaceae
bf7ad91e744ca9ef821623e115c33846	0.9989881	0.7055092	0.9705463	Eubacteriaceae
442c301329744bdb4e54744745b02836	0.9989881	0.346483	0.8658076	Peptostreptococcaceae
47d86d4a1a6bf75977fe65b65660fd91	0.9989881	0.6823936	0.8658076	Clostridiaceae
c1858e9b447d3109a5da8aec4eb4c153	0.9989881	0.5119505	0.8658076	f_Christensenellaceae
c24e90c57a551a138dd36a3663193921	0.9989881	0.720447	0.8658076	uncultured
0205de5ae2a84ec03e4e790220f25fea	0.9989881	0.346483	0.8658076	Butyricicoccaceae
50c1ae203d1f1092d7a47aeda64518e5	0.9997894	0.1613955	0.9173698	Ethanoligenenaceae
e425cd6ab853e65dfceba9433e7faf1e	0.9989881	0.8653158	0.8658076	Ruminococcaceae
f6eb75bbd3ec802e7a412e620d2b3601	0.9989881	0.9380577	0.8658076	[Eubacterium]_coprostanoligenes_group
80d8fc2fb93da3c2a7f31aa48cb5b30d	0.9989881	0.7681701	0.8658076	Oscillospiraceae
c4441181d2378d96e307b1f3e31cbfb6	0.9989881	0.9105043	0.8658076	Lachnospiraceae
Overall by C	dietB	dietP	dietD	Family
	0.7055092	0.3338319	0.0015835	Gastranaerophilales
683e591a876d34cb23e8a68e9fdb8f1a	0.7681701	0.9649944	0.8295355	Coriobacteriales_Incertae_Sedis
4130f21ae0c17450e39a56b46c19737b	0.9380577	0.8228571	0.1452257	Eggerthellaceae
92b4c4b1ddd22276bfdc429a24b3c240	0.9380577	0.8228571	0.9200236	Coriobacteriaceae
f60ff9fcfe17e964a8f857677cd5554a	0.7681701	0.3179652	0.6442406	Atopobiaceae
62df0bf2a96a354cb6311ca343ab768a	0.720447	0.8228571	0.9200236	Bifidobacteriaceae
49450d9736f3e6d2253eea2117bada10	0.5119505	0.3338319	0.9478199	Akkermansiaceae
82016bad4d8e52fd00ca49f66df848e5	0.4866336	0.9588538	0.8295355	Desulfovibrionaceae
a131a5f05f0233f0f1b6539ee20910ac	0.5119505	0.8228571	0.9200236	Enterobacteriaceae
26aa8aa789a10616d1f469f61638b565	0.7055092	0.5516992	0.8295355	Sutterellaceae
2b466ca5dd30c6518367775d70903ca5	0.4866336	0.5129008	0.9200236	Erysipelatoclostridiaceae
a2d22d7ac96caf193608a148f07920e7	0.346483	0.0811229	0.7885345	Erysipelotrichaceae

fd63bc6df4da85bdcb38ace3fa4255ff	0.4866336	0.521938	0.0361621	Streptococcaceae
857d9fcb43282b31d88574dc2160b7e6	0.1613955	0.1062101	0.0474301	Enterococcaceae
ccdaecaebacabdf130765573dbb8d2fc	0.7681701	0.7854776	0.9200236	Acidaminococcaceae
b9c6b2168e9b371e0746e2d064cc3bf6	0.7055092	0.0269715	0.9200236	Veillonellaceae
650ab48868fbc4632633a40d8798bdef	0.4866336	0.0269715	0.9091745	Bacteroidaceae
055eac8ba0268dce24092a805d208e9d	0.9380577	0.8228571	0.8713282	Marinifilaceae
c21c4ed964690297593f43867af9a430	0.7055092	0.644706	0.6986172	Barnesiellaceae
d3e105121385c0368f6ac84df9284d13	0.8924151	0.8228571	0.8713282	Tannerellaceae
13577116fdb95c63a3ebd019575c85ff	0.346483	0.2642463	0.0144271	Rikenellaceae
668a510f30c8545ea672acbf3e20ee1a	0.6823936	0.521938	0.9200236	Anaerovoracaceae
8a9a71b529e55e98b0b76a1ec283d9b7	0.5119505	0.521938	0.0001701	Anaerofustaceae
bf7ad91e744ca9ef821623e115c33846	0.7055092	0.521938	0.8295355	Eubacteriaceae
442c301329744bdb4e54744745b02836	0.346483	0.8228571	0.8147461	Peptostreptococcaceae
47d86d4a1a6bf75977fe65b65660fd91	0.6823936	0.8228571	0.9200236	Clostridiaceae
c1858e9b447d3109a5da8aec4eb4c153	0.5119505	0.7358489	0.9200236	f_Christensenellaceae
c24e90c57a551a138dd36a3663193921	0.720447	0.8228571	0.0471423	uncultured
0205de5ae2a84ec03e4e790220f25fea	0.346483	0.1705828	0.0015389	Butyricicoccaceae
50c1ae203d1f1092d7a47aeda64518e5	0.1613955	0.0269715	0.0474301	Ethanoligenenaceae
e425cd6ab853e65dfceba9433e7faf1e	0.8653158	0.2035423	0.3101352	Ruminococcaceae
f6eb75bbd3ec802e7a412e620d2b3601	0.9380577	0.8228571	0.6986172	[Eubacterium]_coprostanoligenes_group
80d8fc2fb93da3c2a7f31aa48cb5b30d	0.7681701	0.7358489	0.8295355	Oscillospiraceae
c4441181d2378d96e307b1f3e31cbfb6	0.9105043	0.8228571	0.9200236	Lachnospiraceae

Supplementary Figure S1. Correlation hierarchical heatmap of bacteria family abundance and biomarkers. Darker colors represent higher positive correlations, while lighter represent highly negative correlations.

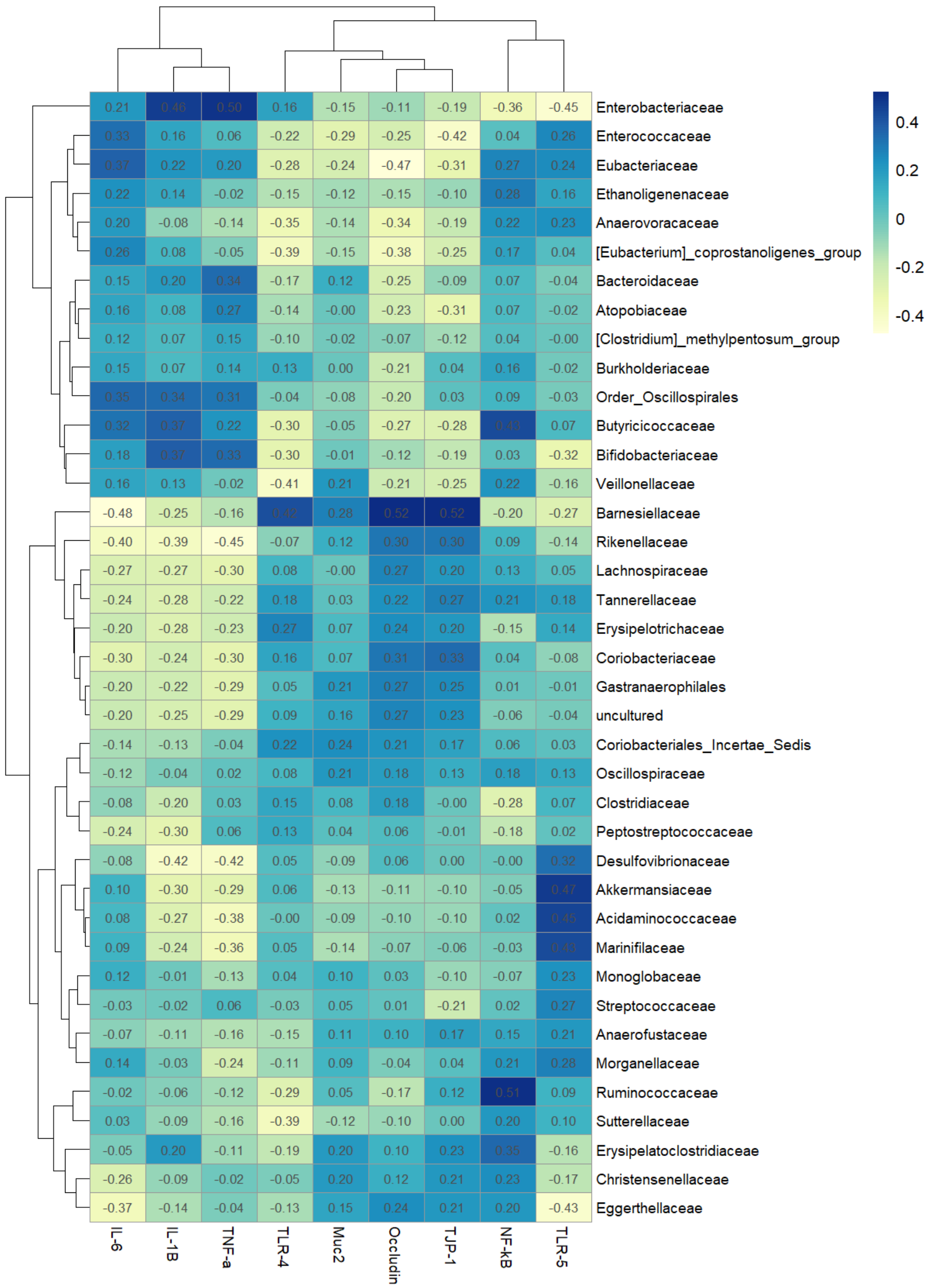

Supplementary Figure S2. Correlation hierarchical heatmap of bacteria family abundance and short chain fatty acids. Darker colors represent higher positive correlations, while lighter represent highly negative correlations.

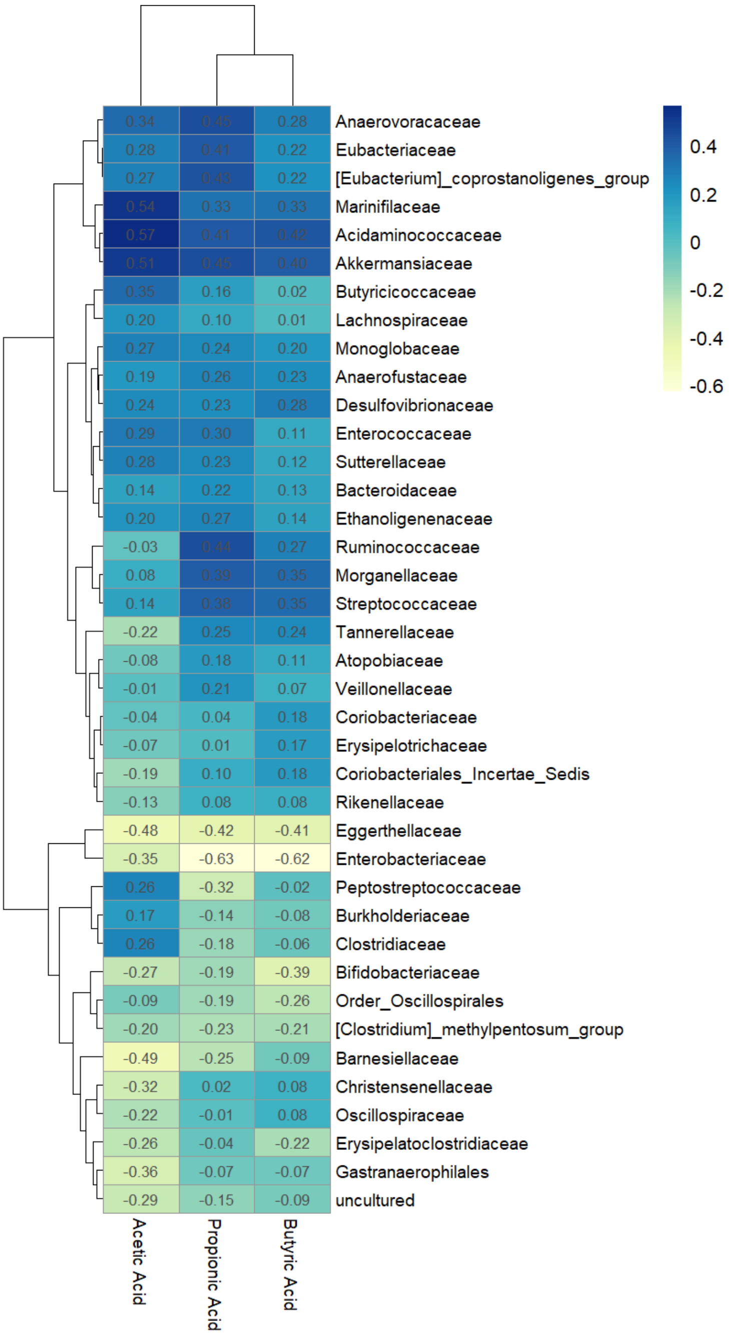

Supplementary Figure S3. Correlation hierarchical heatmap of bacteria family abundance and primary bile acids. Darker colors represent higher positive correlations, while lighter represent highly negative correlations.

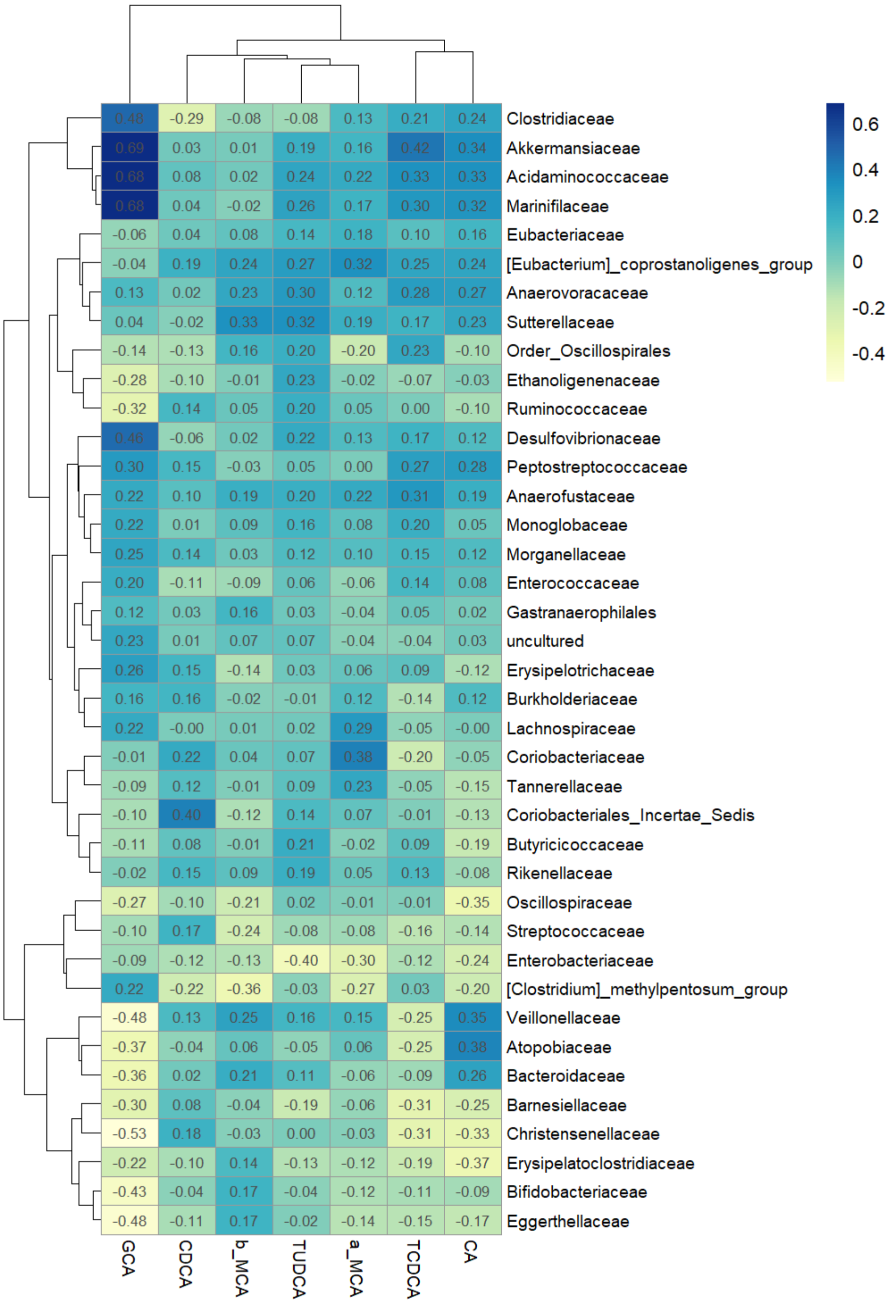

Supplementary Figure S4. Body weight loss / gain (Average percentage ± SEM) per treatment (n=9-12) during experimental period. Diets: purified (P), and 25% inclusion of maize NIL with 3-DFs (B), 3-HFs (C) or 3-DF+3-HF (D).

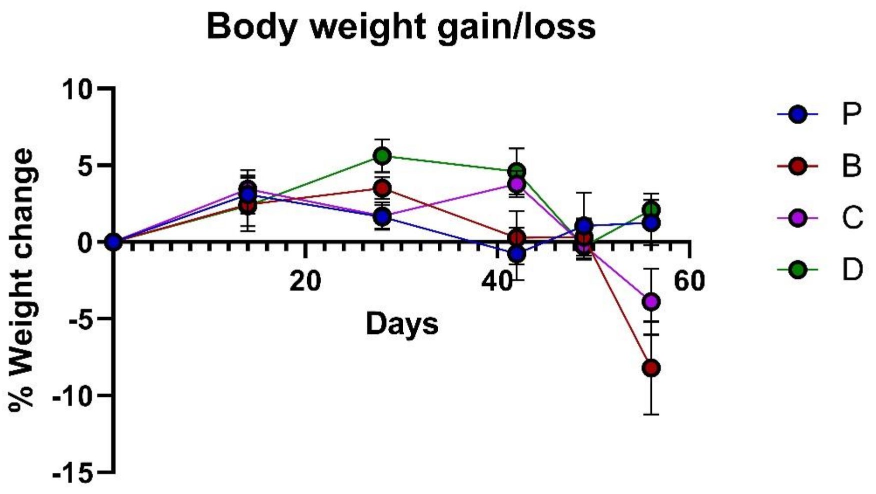

Supplementary Figure S5. Food intake (Average ± CI) per treatment (n=9-12) during experimental period. Diets: purified (P), and 25% inclusion of maize NIL with 3-DFs (B), 3-HFs (C) or 3-DF+3-HF (D).

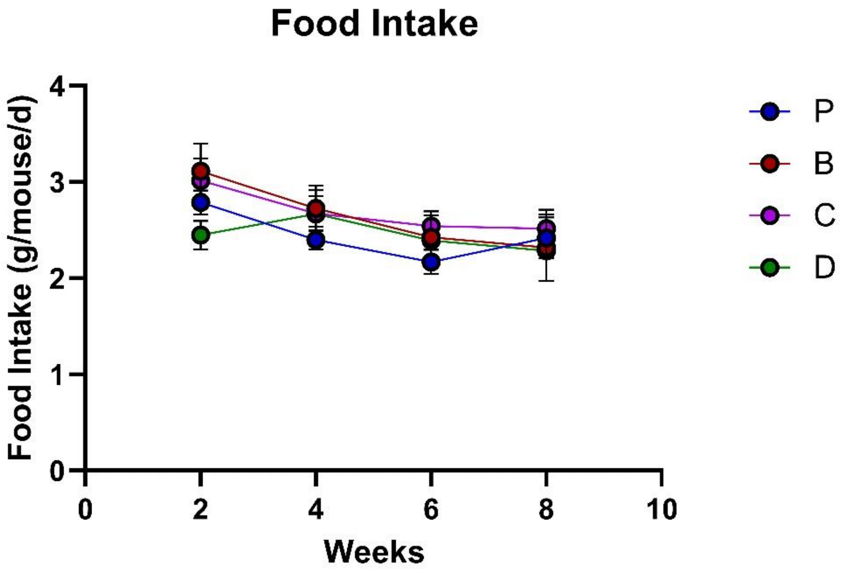

Supplementary Figure S6. Representative histological photomicrographs of hematoxylin and eosin (H&E)-stained paraffin longitudinal section of distal colon tissues. Diets: purified (P), and 25% inclusion of maize NIL with 3-DFs (B) or 3-HFs (C). Human donor: H-1.

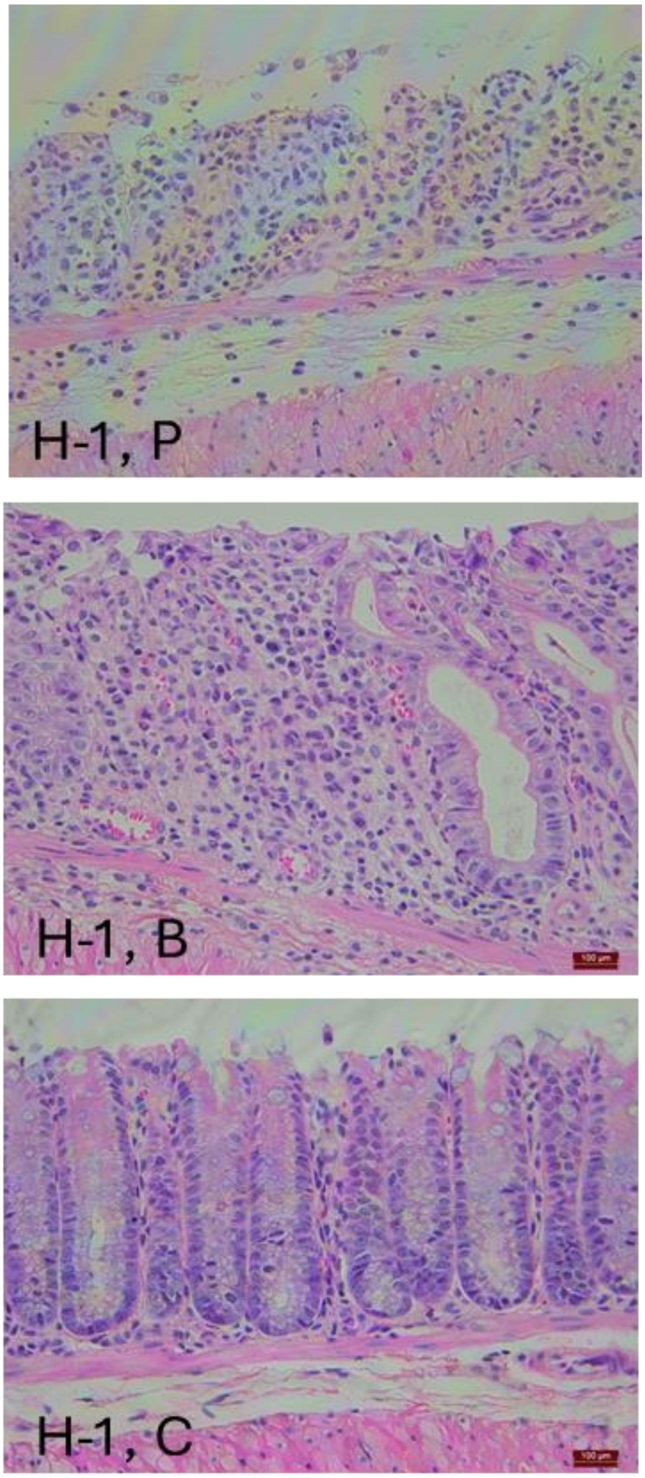

Supplementary Method S1: Conditions of gas chromatography for SCFA analysis. An injection volume of 0.3 µL of SCFA extract was used, with the oven initially set at 50°C for 2 minutes. The temperature was then increased to 70°C at a rate of 10°C/min, followed by ramping up to 85°C at 3°C/min, 110°C at 5°C/min, and 290°C at 30°C/min. The final temperature of 290°C was maintained for 8 minutes. Helium was used as the carrier gas, flowing at a constant rate of 1 mL/min throughout the process.
